# High-efficiency near-infrared optical parametric amplifier for intense, narrowband THz pulses tunable in the 4 to 19 THz region

**DOI:** 10.1038/s41598-022-20622-9

**Published:** 2022-09-29

**Authors:** Meenkyo Seo, Je-Hoi Mun, Jaeuk Heo, Dong Eon Kim

**Affiliations:** 1 Department of Physics and Center for Attosecond Science and Technology, POSTECH, Pohang, 37673 South Korea; 2 Max Planck POSTECH/KOREA Research Initiative, Pohang, 37673 South Korea

**Keywords:** Optics and photonics, Lasers, LEDs and light sources, Optical physics, Optical techniques

## Abstract

Dynamic control of material properties using strong-field, narrowband THz sources has drawn attention because it allows selective manipulation of quantum states on demand by coherent excitation of specific low-energy modes in solids. Yet, the lack of powerful narrowband lasers with frequencies in the range of a few to a few tens of THz has restricted the exploration of hidden states in condensed matter. Here, we report the optimization of an optical parametric amplifier (OPA) and the efficient generation of a strong, narrowband THz field. The OPA has a total conversion efficiency of > 55%, which is the highest value reported to date, with an excellent energy-stability of 0.7% RMS over 3 h. We found that the injection of a high-energy signal beam to a power amplification stage in an OPA leads to high-efficiency and a super-Gaussian profile. By difference-frequency generation of two chirped OPA signal pulses in an organic nonlinear crystal, we obtained a THz pulse with an energy of 3.2 μJ, a bandwidth of 0.5 THz, and a pulse duration of 860 fs tunable between the 4 and 19 THz regions. This corresponds to an internal THz conversion efficiency of 0.4% and a THz field strength of 6.7 MV/cm. This approach demonstrates an effective way to generate narrow-bandwidth, intense THz fields.

## Introduction

Optically excited coherent phonon-driven dynamics is a novel scientific method for ultrafast control of functional electronic, magnetic, and structural properties of quantum materials^1–5^. In particular, the selective excitation of an infrared-active vibration mode to a large amplitude makes it possible to induce collective properties in solids such as insulator–metal transitions^6^, and high-temperature superconductivity^7^. Hence, a powerful THz field with a spectrally narrow-bandwidth is an ideal driving source for these applications.

An organic crystal is one of the promising nonlinear media for generating an intense THz pulse because of its high second-order nonlinear susceptibility and easy phase-matching condition in the THz frequency region^8–11^. In particular, the DSTMS (4-N,N-dimethylamino-4'-N'-methyl-stilbazolium 2,4,6-trimethylbenzenesulfonate) crystal has been proven to produce a strong, narrowband THz field^12–14^.

The DSTMS organic crystal requires near-infrared (NIR) pulses in the wavelength range of 1.3–1.6 μm as pump sources because of its large figure of merit^15^. An intense NIR pulse can be obtained from an optical parametric amplifier (OPA) with compact optical setups. Typically, the conversion efficiency of an OPA using β-barium borate (BBO) crystals pumped by a Ti:Sapphire laser system at kHz repetition rates is about 33–40%^16–18^. It is known that a super-Gaussian spatial distribution is advantageous for efficiently generating THz pulses, compared to a Gaussian profile because the super-Gaussian profile produces a more homogeneous output and is less likely to damage the crystal because of its low intensity^19^ Techniques have been developed such as the shaping of the pump beam profile as flattop or conformal^20,21^, or a dual-chirped optical parametric amplification (DC-OPA) method^22^, to enhance the output pulse energy with a homogeneous beam profile. However, the expense and complexity of such pulse shaping setups may pose a hurdle in practice, in addition to their adverse effects on the output stability.

In this work, high-energy NIR pulses with a super-Gaussian beam profile were employed to generate intense THz pulses in the organic crystal. Here, we report a high conversion efficiency of over 55% in a double-stage OPA by operating the amplification process in a highly-saturated gain regime in a compact layout without adding extra optical components. We show that the injection of high-energy signal pulses in the second stage improves the conversion efficiency even near the saturated gain regime, and the beam profile of the output signal becomes super-Gaussian, deviating from a Gaussian profile. In this high-efficiency operation, the pulse energy stability is in the order of 0.7% RMS for over 3 h with a pulse duration of 33 fs full-width at half-maximum (FWHM). Two output pulses from two identical high-efficiency OPA systems seeded by the same white light continuum, after temporal stretching, are effectively utilized to generate narrowband THz pulses via difference-frequency generation (DFG) in a DSTMS organic crystal. The frequency of THz pulses can be tuned between 4 and 19 THz while maintaining a bandwidth of ~ 0.5 THz. The maximum energy of THz pulses is 3.2 μJ, resulting in a THz conversion efficiency of 0.4% and a peak THz electric field of 6.7 MV/cm.

## Results

### Operation in a highly-saturated gain regime

**Figure 1:**
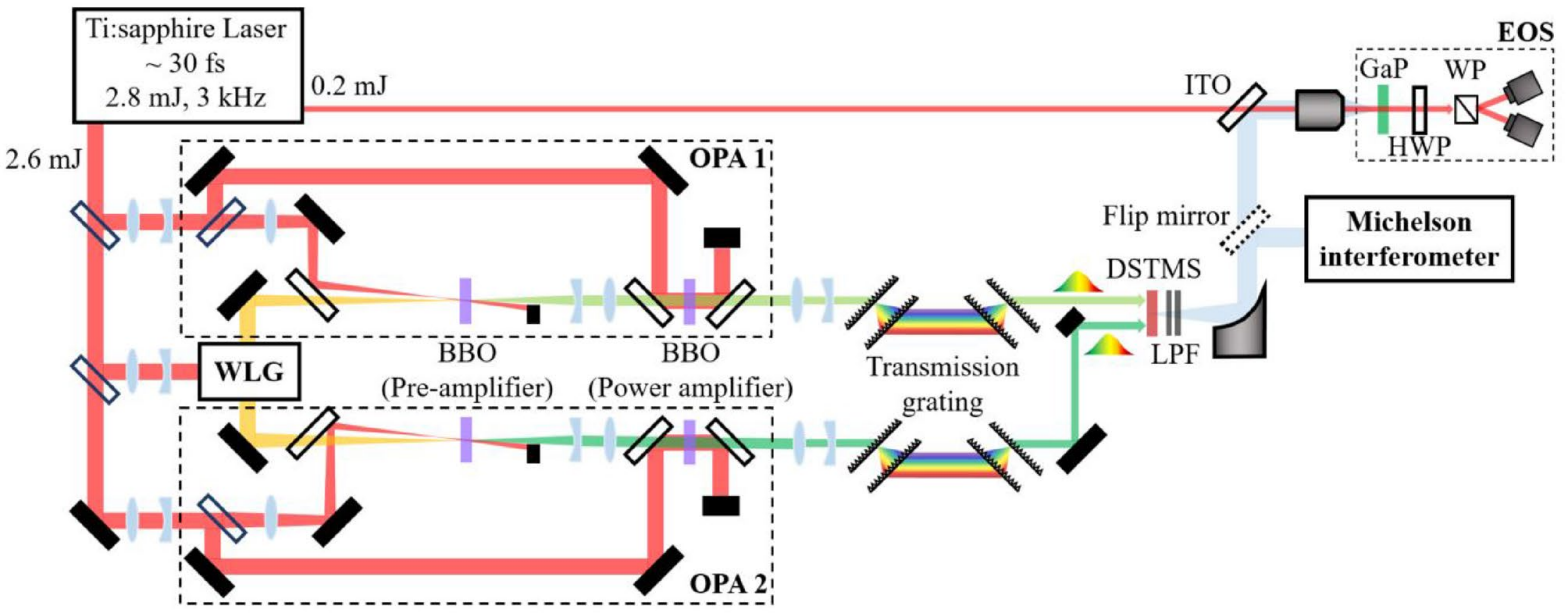
Experimental setup for generating narrowband, strong THz fields, composed of two high-efficiency OPAs. A laser pulse with 2.8 mJ is separated to pump two identical two-stage OPAs seeded by the same white light generation (WLG) system, and to probe for electro-optic sampling (EOS). Each separated beam is down-collimated by a Galilean telescope. The signal output from each OPA is chirped by a pair of high-efficiency transmission gratings and utilized for the DFG process in a DSTMS crystal. The generated THz pulses are extracted by a pair of low pass filters (LPF) and characterized by either interferometric autocorrelation or EOS in a GaP crystal. The EOS setup consists of a half-wave plate (HWP), a Wollaston prism (WP), and two fast photodiodes in a balanced detecting configuration.

**Figure 2:**
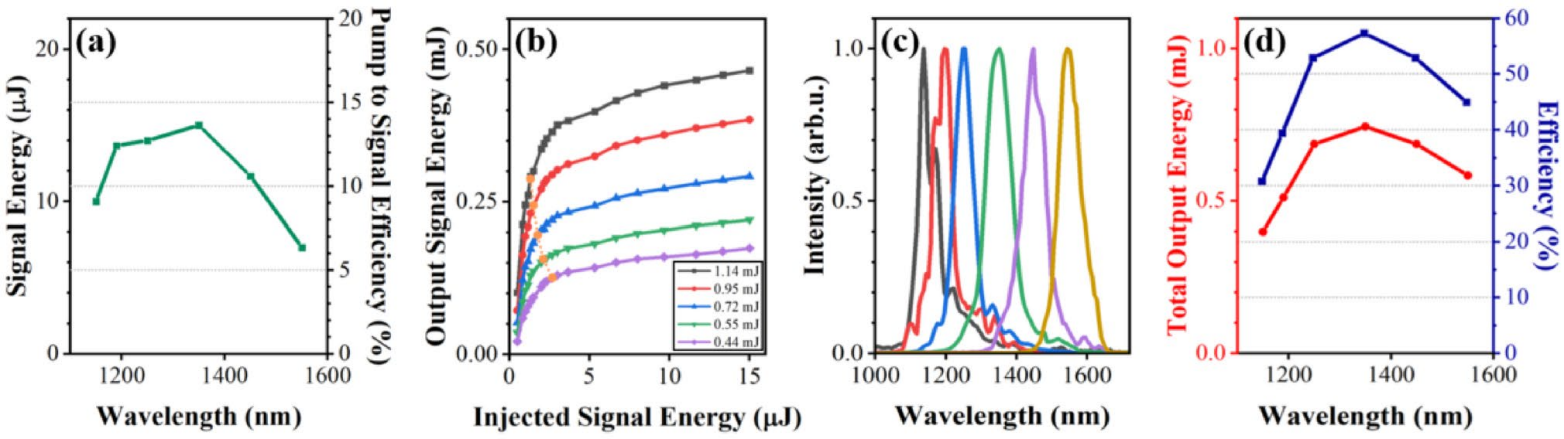
(**a**) Energy of signal pulse (left axis) in the pre-amplifier along with the internal conversion efficiency (right axis). (**b**) Output signal energy as a function of the input signal and pump energy in the power amplifier. (**c**) Spectra of output signals at different wavelengths. (**d**) The total output energy (signal plus idler pulse) (red dots) and the corresponding conversion efficiency (blue squares) for a total pump energy of 1.3 mJ.

The experimental setup for the high-efficiency two-stage OPA is shown in Fig. 1. In contrast to conventional OPA, where a pump-to-signal conversion efficiency of a few percent is obtained in pre-amplifers^23^, in the current setup, a conversion efficiency of about 14% was achieved as shown in Fig. 2a; this represents an increase in efficiency by a factor of almost five; and the signal output energy from the pre-amplifier was 15 μJ at a wavelength of 1350 nm. The key technical points are (1) to adopt a loose-focusing scheme in the white light generation (WLG) process for larger seed energy and (2) to inject a diverging beam into the pre-amplifier so that high pulse energy can be used without damaging the BBO crystal, leading to higher efficiency (see the Methods section). Figure 2b shows that the output signal energy depends on the injected signal energies for a given pump energy. For these measurements, the signal beam diameters were held constant at ~ 5 mm to maintain a match with the pump beam size and to achieve an adequate power density. As the signal energy increases, the amplified signal energy is clearly saturated because of gain depletion. Furthermore, although an injected signal energy of a few microjoules is sufficient to reach the gain saturation region, the output energy still has room to increase, resulting in a higher efficiency for a given pump energy. As the pump energy increases, the output signal energy also increases; however, the minimum input energy for saturation points decreases as displayed by the orange line in Fig. 2b. Hence, the signal beam can be more easily pushed to a high-gain saturation region as the pump energy increases. Note that the saturation points are estimated as the intersection points where the line linearly fitted to the initial linear increase and that fitted to the gain saturation region meet. The wavelength of the signal pulse can be changed by rotating the BBO crystal angle in both the pre-and power amplifiers. The wavelength tunability of the final signal pulse is presented in Fig. 2c. In the wavelength range between 1250 and 1450 nm, the total conversion efficiency (signal + idler) is more than 52% as shown in Fig. 2d for a pump pulse energy of 1.3 mJ. The maximum conversion efficiency of 57% was obtained at a signal wavelength of 1350 nm, where the signal pulse energy was 0.465 mJ.

### Characterization of signal output pulses

**Figure 3:**
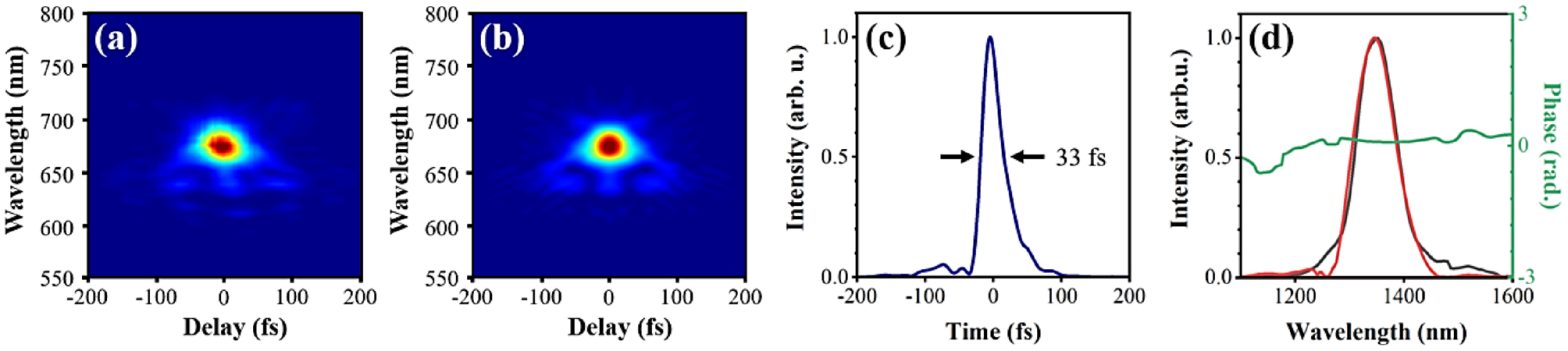
Characterization of the signal outputs at 1350 nm by SHG-FROG. (**a**, **b**) Measured and reconstructed FROG traces. (**c**) Retrieved temporal pulse envelope. (**d**) Retrieved spectrum (solid red line) and phase (solid green line) with the spectrum (solid grey line) measured at the entrance of SHG-FROG.

The final signal outputs are characterized by the second-harmonic generation frequency-resolved optical gating (SHG-FROG) setup to confirm the compressibility. A small fraction of the output beam, after being separated from the pump and idler beam by a dichroic mirror, was taken for SHG-FROG measurements. Figure 3 summarizes the characterization of signal output pulses. The measured pulse duration is 33 fs at a signal wavelength of 1350 nm, which is slightly larger than the input pulse duration of 30 fs. The retrieved pulse was reconstructed from FROG traces using a 256 × 256 grid with a FROG error of 7 × 10^–3^.

**Figure 4:**
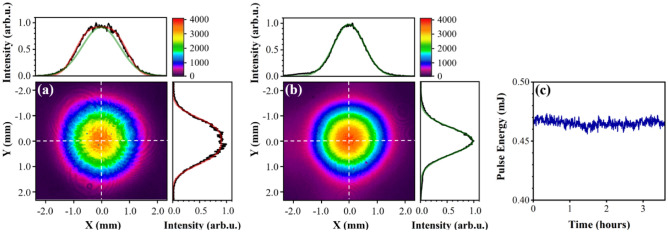
Far-field beam profiles of the second harmonic of the signal beam for an injected signal energy of (**a**) 15 μJ and (**b**) 2.5 μJ. The spatial distributions plotted in the top and right panel are vertical and horizontal cross-sections of the beam profile along with the white dashed line. They are overlaid by a super-Gaussian fitting of order 3 (solid red line) and/or Gaussian fitting (solid green line). The range of color bars is the same for both (**a**) and (**b**). (**c**) Energy stability of final signal output pulses. The fluctuation of 0.7% RMS is maintained at least over 3 h.

Next, we investigated the far-field spatial profile of the OPA output pulses with a beam profiler at a signal wavelength of 1350 nm, where the maximum conversion efficiency was obtained. The beam size of the signal outputs was down-collimated to a diameter of about 2 mm (FWHM). The signal beam was extracted from the co-propagating visible light by two sets of transmission gratings; and then its spatial distribution was measured, using frequency doubling with a BBO crystal, at the position of a DSTMS organic crystal. Figure 4a shows the spatial distribution of the second harmonic of the signal output beam at a wavelength of 1350 nm. The signal beam profile is well-fitted to the super-Gaussian profile of order 3 rather than Gaussian fitting with the same beam diameter at 1/e^2^ as depicted in Fig. 4a. These results are related to the gain depletion in the center of the signal beam^24,25^. The beam profile could be controlled to be Gaussian mode by reducing the signal energy injected into the power amplifier, as shown in Fig. 4b, but this decreased the conversion efficiency from 57 to 39%. For efficient THz generation, we retained the super-Gaussian spatial distribution for signal outputs. Figure 4c presents the energy stability of the signal (1350 nm) pulses. While measuring the energy stability, we also noticed no change in the spectrum and pulse duration of the signal beam. The stability of the signal spectrum has related to the stability of energy. Indeed, the signal spectrum also has excellent long-term stability similar to energy stability. For the pulse duration, there was almost no change before and after the energy stability measurement. An energy fluctuation of 0.7% RMS over 3 h as well as the similar stability of spectrum and pulse duration represents excellent long-term stability.

### Generation of high-energy narrowband THz pulses

**Figure 5:**
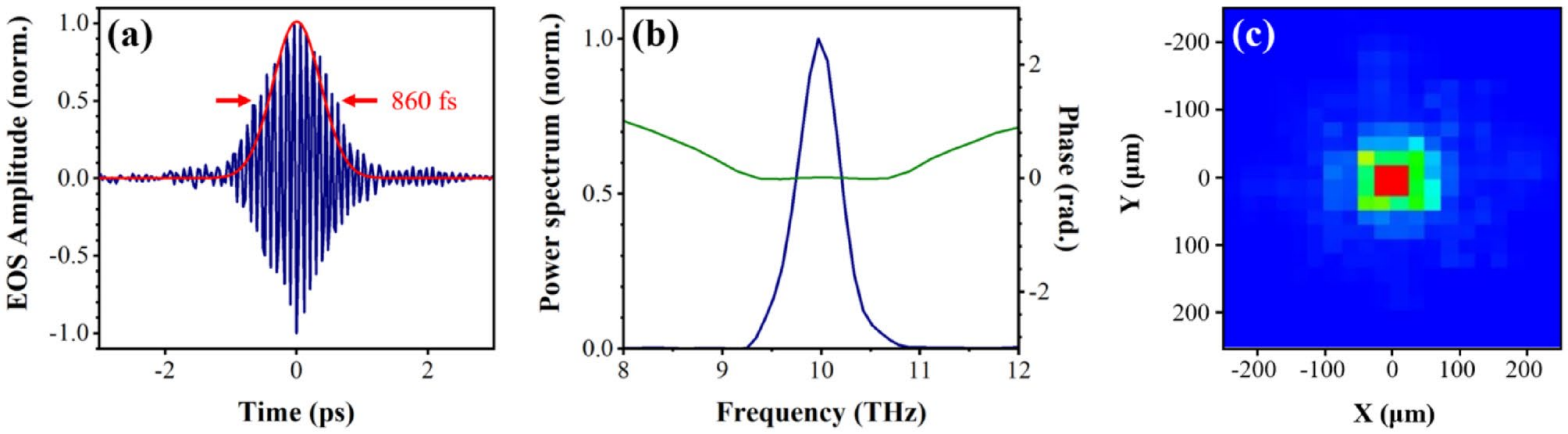
Characterization of the narrowband, phase-stabilized THz pulses generated via DFG. (**a**) THz electric field measured by the EOS method with temporal intensity profile (solid red line) (**b**) The corresponding power spectrum (solid blue line) with spectral phase (solid green line) (**c**) Focused THz beam profile at the EOS crystal position.

**Figure 6:**
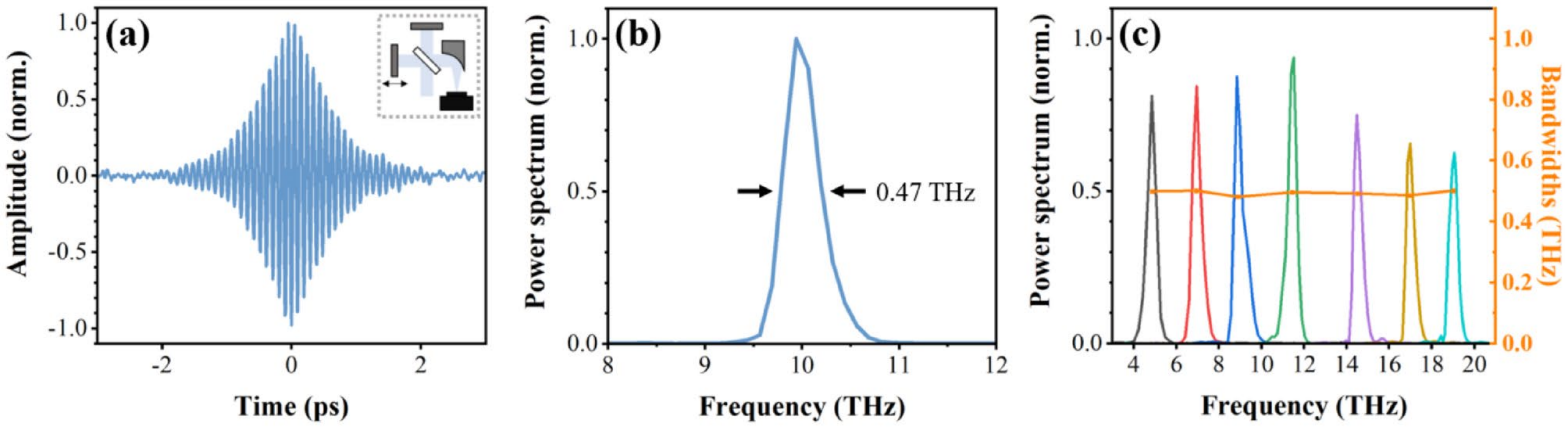
(**a**) Normalized interferogram measured with an interferometer setup (inset) and (**b**) its frequency spectrum, confirming a center frequency of 10 THz and a bandwidth of less than 0.5 THz. (**c**) Tunability and spectral bandwidth (solid orange line) of THz fields, normalized according to the THz field at 10 THz.

In order to efficiently generate the narrowband THz pulses via the DFG process, two signal pulses from OPA1 and OPA2 were temporally stretched to a pulse duration of about 1.2 ps by a high-efficiency transmission grating^26^. Both OPAs carry equal carrier-envelope phase (CEP) offsets because they share the same white light continuum as a seed beam^27,28^. Therefore, the CEP of the generated THz pulses is intrinsically stabilized. Figure 5 shows the characterization of the CEP-stable THz electric field generated by mixing two signal outputs of different wavelengths (1400 and 1470 nm). The signal wavelengths were chosen to minimize the residual third-order dispersion (TOD) from the transmission grating, maintaining a good coherence length region for the DSTMS crystal^29^, because any difference in TOD between the signal pulses for the DFG results in a lower THz conversion efficiency and broader bandwidth^30^. For the electro-optic sampling (EOS) measurement, the generated THz pulse energy of 3.2 μJ was attenuated by a factor of 50 with a pair of THz polarizers to avoid the probe polarization over-rotation and high-order nonlinear effect due to high peak electric field^28^. The THz pulses were then focused by a reflective objective (Numerical aperture = 0.52) onto the electro-optic detection crystal. The THz pulse duration of 860 fs is estimated from the EOS trace as depicted by the solid red line in Fig. 5a. The corresponding spectrum with the almost flat phase plotted in Fig. 5b shows a center frequency of 10 THz and a bandwidth of less than 0.5 THz. Figure 5c displays a focal spot diameter of 75 μm (FWHM) measured with a THz microbolometer camera after further attenuating the THz pulse energy to avoid the saturation of the image, resulting in a peak electric field of 6.7 MV/cm and a peak intensity of 60.6 GW/cm^2^ without attenuation. These results indicate that our high-efficiency OPA can produce the intense THz pulses with a THz conversion efficiency of 0.4% with respect to NIR pump energy. The GaP crystal has been used for EOS measurement for lower frequencies than 7 THz due to the mismatch between the THz phase velocity and the optical group velocity of the laser pulse, and strong phonon absorption in higher frequencies. The velocity mismatches have been evaluated by the absolute magnitude of the geometric response function, a quantitative measure of velocity mismatches^31^: there would be no EOS signal at given frequencies if the geometric response function vanishes at those frequencies. Because the response function of a 0.1 mm thick GaP crystal has finite values for up to 11 THz^31^, the EOS measurement is still usable for the narrowband (< 0.5 THz) THz pulses at 10 THz. With respect to the absorption issue, the absorption bandwidth of GaP crystal at 11 THz is less than 0.5 THz^31^. Since the bandwidth of our THz pulse at 10 THz is also less than 0.5 THz, the absorption effect by a thin 0.1 mm thick GaP crystal would be minimal. To make sure that our EOS measurement is correct, we also performed the first-order autocorrelation based on the Michelson interferometer as shown in Fig. 6a. The corresponding spectrum, as displayed in Fig. 6b, shows a center frequency of 10 THz and a bandwidth of 0.47 THz (FWHM). Figures 5b and 6b clearly show the generation of the frequency difference between the two signal pulses of 214.1 THz and 203.9 THz. The good agreement between Figs. 5 and 6 confirms the reliability of our EOS results. In a similar manner, THz pulses with other center frequencies were efficiently generated by simultaneously tuning the wavelength of the two signal pulses. Figure 6c presents the output power spectra at selected THz frequencies covering the spectral region between 4 and 19 THz. The bandwidth of about 0.5 THz was maintained during these measurements. The pulse energy varied between 2.0 and 3.2 μJ. Even for the strong absorption at 8 and 12 THz in the DSTMS crystal^32^, reasonable outputs were obtained at 9 and 11 THz. These high pulse energies are sufficient to reach a field strength at the MV/cm level.

## Discussion

We present a method for optimizing the OPA to efficiently generate narrowband, high-energy, CEP-stable THz pulses in a compact setup. The OPAs have a maximum conversion efficiency of 57% with an excellent long-term stability, producing a super-Gaussian beam profile. It is demonstrated that the injection of high-energy signal pulses into a power amplification stage can enhance the conversion efficiency while the spatial profile of the output signal beam becomes flattened. It was shown that two signal output pulses from two identical OPAs, after being temporally stretched, efficiently generate narrowband THz pulses in the 4–19 THz range via DFG in an organic crystal. A THz pulse energy of up to 3.2 μJ has been achieved, which corresponds to a field strength of 6.7 MV/cm. With a limited pump energy, the optimization for a high THz conversion efficiency is necessary for high-energy THz pulses. Furthermore, the stability of output pulses is also a crucial parameter in the application to actual experiments. In this regard, our results pave the way to a powerful method for improving the conversion efficiency of both OPA and THz generation with high stability. Also, this system can be scaled up to even higher pulse energies with a high-energy pump laser system by properly adjusting the beam size in all of the nonlinear processes. Such a strong-field narrowband THz pulse opens new possibilities for the advanced manipulation of collective electronic properties in condensed matter by selective excitation of low-energy modes.

## Method

### Pump laser system

A carrier-envelope phase-stabilized, multi-pass Ti:sapphire amplifier was used as a pump source to provide 30 fs laser pulses with an energy of 2.8 mJ at a repetition rate of 3 kHz and a central wavelength of 780 nm (Femtopower™, Femtolasers). The pump beam was actively stabilized by a beam stabilizer before it was sent to the experimental setup. A pump energy of ~ 2.6 mJ was injected into two double-stage OPAs seeded by the same WLG system. The pump energy for each OPA was about 1.3 mJ and a small energy of 15 μJ was used for WLG. The remainder of the energy (~ 0.2 mJ) was directed to the EOS measurement setup.

### Double-stage OPA

For the WLG, the input beam size was down-collimated by a Galilean telescope to a spot with a diameter of ~ 2.5 mm for loose-focusing. The input beam was focused by a lens (f = 15 cm) into a 2 mm thick sapphire plate. An iris was placed before the focusing lens to control the intensity on the sapphire plate, resulting in a single-filament white-light continuum (WLC) with an excellent radial profile. This loose-focusing scheme and careful alignment allow one to increase the available seed energy for the pre-amplifier without inducing damage on the sapphire plate. The WLC was split by a beam splitter for seeding OPA1 and OPA2; its beam diameter and divergence were controlled by an achromatic lens (f = 5 cm) to match the pump beam size in each pre-amplifier. The pre-amplifier has a non-collinear geometry with an angle of ~ 2.5° between the pump and the seed beam. The pump beam with a pulse energy of 0.11 mJ was focused 16 cm before a type-II BBO crystal (2.5 mm thick, θ = 28°) by a lens (f = 75 cm). This corresponds to an intensity of 240 GW/cm^2^ at the BBO crystal. Such a diverged pump beam counteracts the self-focusing effect in the BBO crystal so that a high pump energy can be applied without inducing damage to the BBO crystal. The output signal beam was expanded and collimated to a beam ~ 5 mm in diameter. In the power amplifying stage, a collinear geometry was used between the pump and injected signal beam. The pumping energy was 1.14 mJ, resulting in an intensity of 230 GW/cm^2^ on the second type-II BBO crystal (3.0 mm thick, θ = 28°). The final signal outputs were separated from the pump and idler beam by dichroic mirrors. Another identical double-stage OPA was built. Two signal outputs from each OPA were down-collimated to a spot ~ 2.0 mm in diameter (FWHM) and chirped as much as ~ 1.2 ps by a high-efficiency transmission grating (T-940C, LightSmyth™) to keep the pulse intensity below 150 GW/cm^2^ and thereby avoid damaging the DSTMS crystal and suppressing coherent THz emission by optical rectification.

### Efficient narrowband THz pulse generation

Two chirped NIR pulses with a super-Gaussian beam profile were utilized in the DFG process to effectively generate narrowband THz pulses in a 500 μm thick DSTMS crystal. For efficient DFG, a type-0 phase-matching condition was adopted with a quasi-collinear geometry of < 0.1°. Furthermore, for all generated THz pulse frequencies, the center wavelength of the NIR pulses was tuned to minimize the TOD difference between them while maintaining a favorable coherence length in the DSTMS crystal. The generated THz pulses were separated from the residual NIR beam by a pair of 20 THz low-pass filters and characterized with the Michelson interferometer and EOS measurements. A 100 µm thick GaP crystal was used for EOS measurement with a gating pulse of 30 fs. The gating beam after the motorized delay line collinearly propagated with THz pulses through an indium tin oxide (ITO) coated beam splitter, and was focused on the GaP crystal by a reflective objective. Note that the THz pulse energy was attenuated before the ITO beam splitter by a pair of THz polarizers (Tydex, POL-PP-CA25-OD40-T8). The electro-optic signal induced by the THz field was detected in the balanced detecting configuration with a half-wave plate and a Wollaston prism. The THz pulse energy was measured by a pyroelectric detector (THZ9B-BL-DA-D0, Gentec). The beam profile of THz pulses was measured by a microbolometer camera (Rigi uncooled, Swiss Terahertz LLC).

## Data Availability

The datasets generated and analyzed during the current study are available from the corresponding author on reasonable request.
